# Omental Serous Cystadenofibroma Mimicking Disseminated Malignancy: A Rare Pathology

**DOI:** 10.14740/jmc5316

**Published:** 2026-04-29

**Authors:** Chibuike Ezeibe, Loren Walwyn-Tross, Dusan Perisic

**Affiliations:** a Department of Obstetrics and Gynecology, Saint Peter’s University Hospital, New Brunswick, NJ, USA; b Division of Minimally Invasive Gynecologic Surgery, JFK University Medical Center, Edison, NJ, USA

**Keywords:** Cystadenofibroma, Ovarian neoplasms, Omental mass, Benign ovarian tumors, Multicentric lesions

## Abstract

Serous cystadenofibromas are rare benign epithelial ovarian tumors. They account for < 2% of benign ovarian tumors and typically affect individuals aged 15–65. In rare cases, these tumors present with scattered pelvic lesions resembling malignant dissemination. A 68-year-old female presented with pelvic pain. Pelvic sonogram revealed a 6 cm mass anterior to the cecum and bilateral cystic ovarian lesions measuring approximately 5 and 4 cm on the right and left, respectively. Subsequent pelvic magnetic resonance imaging (MRI) confirmed multicystic complex ovarian masses and a 6 cm multicystic mass along the cecum. Colonoscopy revealed no intrinsic rectal or sigmoid disease. She was subsequently referred to minimally invasive gynecology surgery and colorectal surgery for further management. Tumor markers and a ROMA panel were within normal limits. Computed tomography (CT) of abdomen/pelvis redemonstrated a multilobulated, cystic mass in the right lower quadrant with adjacent satellite lesions and bilateral cystic adnexal masses. Surgical intervention included robotic-assisted total laparoscopic hysterectomy, bilateral salpingo-oophorectomy, extensive lysis of adhesions, and infracolic omentectomy. Intraoperative findings revealed dense adhesions between the omentum, right adnexa, pelvic sidewall, and cecum. Frozen section revealed bilateral ovarian cystadenofibroma and omental tissue with benign cystic lesions resembling serous cystadenofibroma. Postoperatively, the patient had an uneventful recovery. Serous cystadenofibromas are associated with a favorable prognosis after complete resection of the tumor. The use of preoperative imaging and intraoperative frozen sections can help guide appropriate surgical management. However, these tumors pose diagnostic challenges due to their complex nature of adnexal masses with solid and cystic elements, often leading to misdiagnosis of malignancy. In rare cases, the presence of multiple lesions may be more consistent with multicentric development or independent primary lesions rather than true dissemination. Although malignancy must be carefully excluded, disseminated benign serous cystadenofibroma implants may radiologically and intraoperatively mimic peritoneal carcinomatosis, underscoring the importance of a broad differential diagnosis to guide intraoperative judgment and avoid overtreatment.

## Introduction

Ovarian cystadenofibromas are rare, benign, serous ovarian tumors, representing approximately 1.7% of all benign ovarian tumors, and are commonly seen in women aged 15–65 years [1–4]. Clinical presentation is typically nonspecific, manifesting as pelvic and/or abdominal pain, bloating, pelvic pressure, back pain, or constipation [1–4]. Physical examination may reveal a palpable adnexal mass. Radiologic imaging, including pelvic ultrasound and magnetic resonance imaging (MRI), typically demonstrates cystic adnexal masses with septations, solid elements, and/or papillary projections [4]. The presence of these solid components may lead to misdiagnosis as malignant during initial evaluation [5].

Management of cystadenofibromas typically involves surgical resection, with minimally invasive approaches preferred when feasible to reduce perioperative morbidity. However, preoperative differentiation from malignant ovarian tumors remains challenging due to overlapping imaging characteristics. Intraoperative frozen section analysis can be instrumental in guiding the extent of surgery and avoiding unnecessary oncologic staging procedures.

In rare instances, cystadenofibromas may present with multifocal or extraovarian lesions that mimic disseminated malignancy [3]. In this case report, we describe a patient with bilateral ovarian cystadenofibromas and omental involvement, highlighting the diagnostic challenges and surgical considerations associated with this unusual presentation.

## Case Report

This case begins with a 68-year-old female who presented to her primary gynecologist for complaints of pelvic pain. Her past medical history was significant for essential hypertension, hyperlipidemia, and hypothyroidism (due to thyroidectomy). All her co-morbidities were stable on medication. Apart from her thyroidectomy, her surgical history was significant for bilateral tubal ligation, cholecystectomy, and umbilical hernia repair.

**Figure 1:**
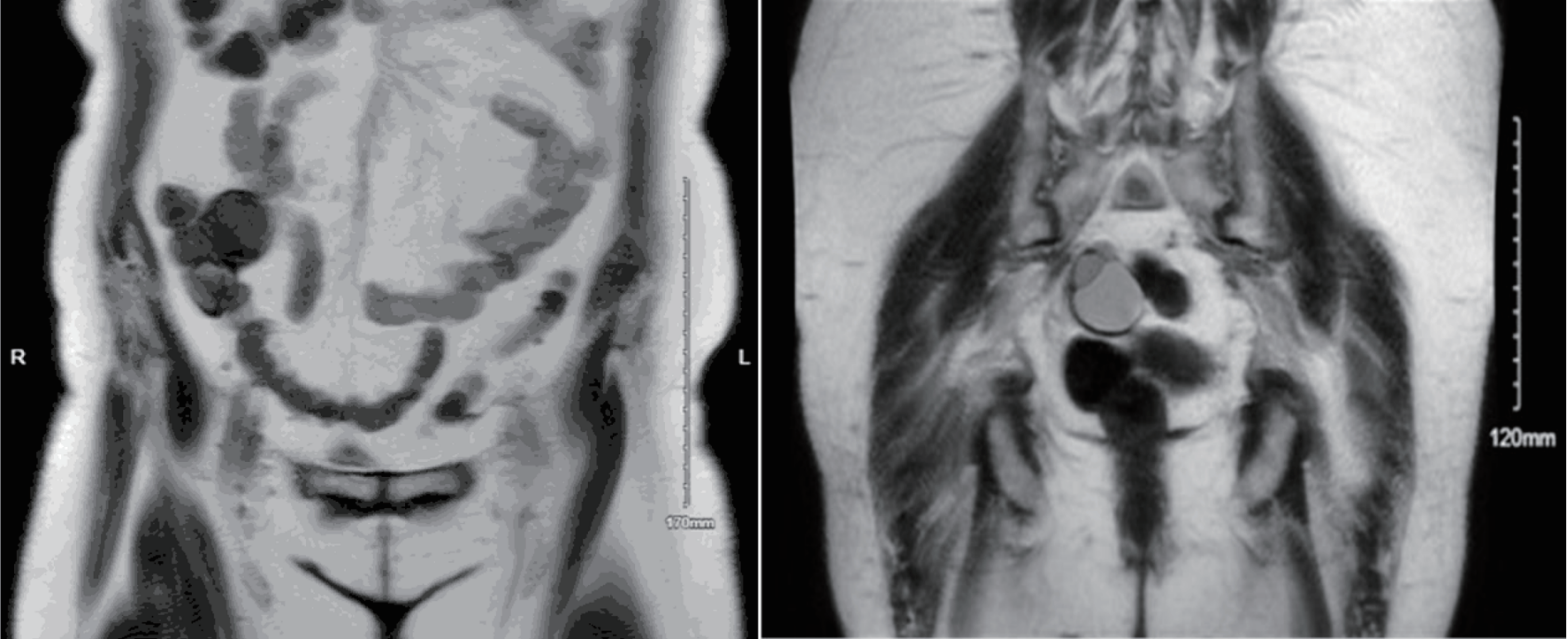
Coronal MRI images demonstrate an omental cystadenofibroma and an ovarian cystadenofibroma. The omental lesion appears as a well-circumscribed cystic mass within the anterior abdominal cavity, consistent with a benign omental implant, while the ovarian cystadenofibroma is located lower in the pelvis adjacent to the uterus, showing mixed cystic and solid components typical of epithelial origin. MRI: magnetic resonance imaging.

In March 2020, she initially underwent a pelvic ultrasound that showed a 5.3 × 5.3 × 4.7 cm multiseptated cystic lesion on the right ovary and a 2.8 × 2.1 × 2.8 cm septated cyst on the left ovary. MRI of pelvis as well as further gynecologic evaluation were recommended. In April 2021, she presented to an adjacent hospital system where she was evaluated in the emergency room for lower abdominal pain. A computed tomography (CT) scan of the abdomen and pelvis was performed. The CT scan showed a 5.0 × 3.6 × 4.3 cm low-density nodule posterior and rightward of the uterus, associated with the right ovary and a 3.0 × 4.2 × 3.0 cm low-density nodule anterior and leftward of the uterus, associated with the left ovary. Additionally, there was a 9 mm enhancing nodule in the posterior cecal wall, not previously seen on initial imaging, as well as a multi-lobulated multicystic complex mass anterior to the inferior cecum. As a result, she was later sent for a pelvic MRI which still showed bilateral ovarian pathology seen on previous imaging. The MRI (Fig. 1, key image) showed complex multicystic changes in the right ovary measuring 3.4 × 3.3 × 3.4 cm with multiple thin septations. The left adnexa showed similar complex multicystic changes (3.6 × 1.8 × 2.1 cm with multiple thin septations). The multicystic omental mass was still visualized along the medial aspect of the cecum measuring approximately 6.2 × 4.0 × 3.6 cm with a dominant septation traversing the mass. The cystic lesion did not seem to originate from sigmoid or uterus. She then underwent a colonoscopy which did not show any intrinsic rectal or sigmoid disease.

**Figure 2:**
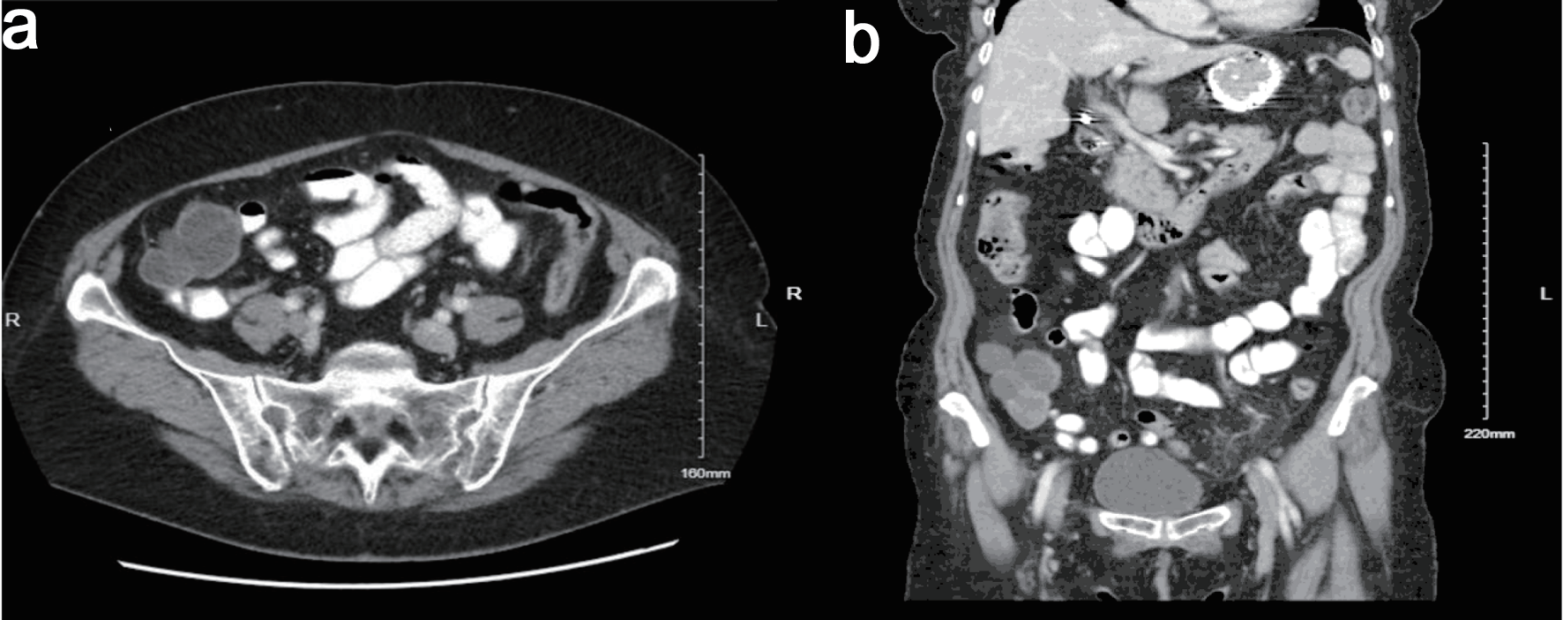
Axial (a) and coronal (b) computed tomography (CT) images demonstrating an omental cystadenofibroma. The lesion appears as a well-circumscribed cystic mass within the anterior abdomen, consistent with a benign omental implant. The coronal view (b) provides a complementary perspective showing the lesion’s relationship to adjacent bowel loops and pelvic organs, further emphasizing its benign, non-infiltrative appearance.

After this initial workup, her primary gynecologist referred her to a minimally invasive gynecology surgeon for further management. A colorectal surgeon was also consulted due to the cecal mass. After consultation with both surgeons, a plan for surgery was made. The patient was recommended to undergo a combined procedure of total abdominal hysterectomy, bilateral salpingo-oophorectomy, and excision of the pelvic mass, with colorectal surgery on hand for possible bowel resection with primary anastomosis. Given the uncertain origin of the omental mass, as well as the persistent presence of bilateral multicystic ovaries masses, preoperative tumor markers including a ROMA panel, carcinoembryonic antigen (CEA), and carbohydrate antigen 19-9 (CA 19-9) were obtained to aid in risk stratification and to help distinguish between gynecologic and gastrointestinal sources prior to surgical intervention. All results were within normal limits. A repeat CT scan of the abdomen and pelvis was recommended to assess if mass remained persistent prior to proceeding with surgery. The repeat CT scan (Fig. 2) showed a 4.0 × 5.8 × 7.2 cm mass in the right lower quadrant mesentery abutting the distal ileum and the cecum. It also redemonstrated cystic lesions in bilateral adnexa, right greater than left, that did not appear significantly increased in size from prior imaging.

The patient returned to minimally invasive gynecologic surgery and colorectal surgery services to review imaging results and finalize the details and plan for surgery. A request was made to Surgical Oncology for a possible intra-operative consultation if frozen pathology was malignant and surgical staging was needed. After preoperative medical clearance with an electrocardiogram, and subsequent transthoracic echocardiogram and exercise stress test, the patient underwent operative laparoscopy, extensive lysis of pelvic adhesions, and infracolic omentectomy with a large mass found in the omentum that was attached to the cecum and the mesentery of the cecum. The specimen was sent for frozen section and showed a benign cystadenofibroma. Given the benign pathology, surgical staging was not indicated, and the decision was made to proceed with the case in a minimally invasive approach. A robotic-assisted total laparoscopic hysterectomy and bilateral salpingo-oophorectomy was performed. The final pathology showed a uterus with atrophic endometrium and adenomyosis, as well as bilateral fallopian tubes with no significant pathologic changes. Final pathology confirmed bilateral ovarian cystadenofibromas as well as multiple cystic lesions (exact number not specified by pathology) within the infracolic omentum, consistent with benign serous cystadenofibroma-like lesions. Her surgery was deemed uncomplicated, and she had an uneventful postoperative course with resolution of her pain.

## Discussion

Cystadenofibromas are benign epithelial ovarian tumors characterized by both cystic and fibrous stromal components and are associated with a favorable prognosis after complete surgical resection. Although benign in nature, these tumors can present significant diagnostic challenges, often mimicking the radiologic features of malignant ovarian neoplasms. These features include solid components, septations, and papillary projections, and often lead to initial suspicion of malignancy, particularly in postmenopausal patients [3, 5–8].

This case highlights an unusual presentation of ovarian cystadenofibroma with additional omental lesions, raising concern for disseminated intra-abdominal malignancy. The presence of a multilobulated omental mass with adjacent lesions further complicates the clinical picture. However, normal tumor markers and intraoperative frozen section findings were critical in guiding appropriate surgical management and avoiding unnecessary oncologic staging procedures.

Clinicians should consider cystadenofibroma in the differential diagnosis of complex adnexal masses, particularly when tumor markers are within normal limits and imaging demonstrates well-circumscribed lesions without clear invasive features. Peritoneal lesions that resemble serous cystadenofibromas of the ovary are rare but have been previously described [3]. Because the peritoneal mesothelium shares a common origin with the ovaries and fallopian tubes, ovarian tumor-like lesions may occur in the peritoneum. It is thought that these lesions arise from endosalpingiosis and may form cystic mass-like lesions anywhere in the peritoneum, omentum, or fallopian tubes. In this setting, the presence of multiple lesions may be more consistent with multicentric development or possibly independent primary lesions rather than true dissemination, given the benign nature of cystadenofibromas [3].

Management typically involves surgical excision, with minimally invasive approaches preferred when feasible. Intraoperative frozen section plays a pivotal role in determining the extent of surgery, particularly in cases where malignancy cannot be excluded preoperatively. Recognition of this entity is essential to prevent overtreatment and avoid unnecessary radical procedures, especially in cases that mimic peritoneal carcinomatosis [3, 5–8]. Histopathologic microphotographs were not available for inclusion, which represents a limitation of this report.

### Conclusions

Often misinterpreted on imaging, the presence of ovarian cystadenofibromas with omental or peritoneal implants highlights a rare case of benign pathology mimicking disseminated malignancy. Armed with the knowledge of this atypical presentation, clinicians can avoid misdiagnosis. Recognition of its characteristic imaging and pathologic features can help guide appropriate surgical management and patient counseling. Although malignancy must be carefully excluded, this case illustrates that disseminated benign serous cystadenofibroma implants may radiologically and intraoperatively mimic peritoneal carcinomatosis, underscoring the importance of a broad differential diagnosis to guide intraoperative judgment and avoid overtreatment.

## Data Availability

No datasets were generated or analyzed during the current study. All relevant clinical information is included within the article.
